# Proteomics reveals the significance of vacuole Pi transporter in the adaptability of *Brassica napus* to Pi deprivation

**DOI:** 10.3389/fpls.2024.1340867

**Published:** 2024-03-25

**Authors:** Bei Han, Junjun Yan, Tao Wu, Xinyu Yang, Yajie Wang, Guangda Ding, John Hammond, Chuang Wang, Fangsen Xu, Sheliang Wang, Lei Shi

**Affiliations:** ^1^ National Key Laboratory of Crop Genetic Improvement, Huazhong Agricultural University, Wuhan, China; ^2^ ZJU-Hangzhou Global Scientific and Technological Innovation Center, Zhejiang University, Hangzhou, Zhejiang, China; ^3^ Microelement Research Center, College of Resources & Environment, Huazhong Agricultural University, Wuhan, China; ^4^ School of Agriculture, Policy and Development, University of Reading, Reading, United Kingdom

**Keywords:** *Brassica napus*, BnPHT5, *1a*, vacuolar Pi transporter, Pi homeostasis, proteome

## Abstract

Vacuolar Pi transporters (VPTs) have recently been identified as important regulators of cellular Pi status in *Arabidopsis thaliana* and *Oryza sativa*. In the oil crop *Brassica napus*, *BnA09PHT5;1a* and *BnC09PHT5;1a* are two homologs of *AtPHT5;1*, the vacuolar Pi influx transporter in *Arabidopsis*. Here, we show that Pi deficiency induces the transcription of both homologs of *PHT5;1a* genes in *B. napus* leaves. *Brassica PHT5;1a* double mutants (DM) had smaller shoots and higher cellular Pi concentrations than wild-type (WT, Westar 10), suggesting the potential role of *BnPHT5;1a* in modulating cellular Pi status in *B. napus*. A proteomic analysis was performed to estimate the role of *BnPHT5;1a* in Pi fluctuation. Results show that Pi deprivation disturbs the abundance of proteins in the physiological processes involved in carbohydrate metabolism, response to stimulus and stress in *B. napus*, while disruption of *BnPHT5;1a* genes may exacerbate these processes. Besides, the processes of cell redox homeostasis, lipid metabolic and proton transmembrane transport are supposed to be unbalanced in *BnPHT5;1a* DM under the -Pi condition. Noteworthy, disruption of *BnPHT5;1a* genes severely alters the abundance of proteins related to ATP biosynthesis, and proton/inorganic cation transmembrane under normal Pi condition, which might contribute to *B. napus* growth limitations. Additionally, seven new protein markers of Pi homeostasis are identified in *B. napus*. Taken together, this study characterizes the important regulatory role of *BnPHT5;1a* genes as vacuolar Pi influx transporters in Pi homeostasis in *B. napus*.

## Introduction

Phosphate (Pi) is a principal component of many macromolecules, such as nucleic acids, phospholipids and ATP, and is essential for plant growth and development (Raghothama, 2000; White and Hammond, 2008). Although high amounts of Pi can be present in soil, it is easily fixed by metal cations to form insoluble compounds or bound in organic forms (Kochian et al., 2004). This can lead to the low Pi availability in the soil and subsequently low Pi uptake potential by plants, leading to Pi deficiency in plants. For crop plants, farmers often apply Pi-containing fertilizers although natural phosphate (Pi) resources are limited and non-renewable, and excessive application of Pi fertilizers also aggravates environmental pollution (Shen et al., 2011). For the wild-type plants, viability depends on how well plants respond to Pi stress. Vacuole is the largest organelle in plant cells. The vacuole Pi separation of plant cells is the main mechanism used by plants to adapt to external fluctuations in Pi availability (Shitan and Yazaki, 2013). If the Pi taken up by the plant is higher than the demand from the cytoplasm, the cytoplasm Pi will be transported to the vacuole for storage to prevent Pi toxicity (Versaw and Garcia, 2017). When the external Pi supply is interrupted, the Pi in the vacuole will be transported to the cytoplasm to ensure cells’ normal physiological function (Shitan and Yazaki, 2013).

Vacuole Pi transporters have been cloned and functionally identified including vacuole efflux type VPE proteins in rice (Xu et al., 2019) and vacuole influx type PHT5 in rice, *Arabidopsis* and *Brassica napus* (Wang et al., 2012; Liu et al., 2015; Wang et al., 2015; Liu et al., 2016; Han et al., 2022). Loss of function of OsVPE1 and OsVPE2 genes causes vacuole accumulation of Pi in rice cells and hinders growth (Xu et al., 2019). AtPHT5s transport Pi into the vacuole and disruption of PHT5s in *Arabidopsis* impairs physiological Pi status leading to increased cellular Pi, decreased vacuole Pi, retarded growth and Pi toxicity in flowers (Liu et al., 2015, 2016; Luan et al., 2019). In B. napus, members of the BnPHT5;1 subfamily are the closest homologs to AtPHT5;1 (Han et al., 2022). Similar physiological Pi status, Pi transport activity and growth performance are reported in the BnPHT5;1b double mutants as observed in Atpht5 Arabidopsis (Han et al., 2022). These studies demonstrate that vacuoles are crucial for the dynamic balance of physiological Pi through multiple tonoplast-embedded Pi transporters to optimize plant growth under different Pi availability (Luan et al., 2019).

Mass spectrometry-based proteomics analyses have been widely conducted to identify proteins that respond to Pi stress conditions in various crops including *B. napus* (Li et al., 2008; Yao et al., 2011; Feng et al., 2012; Zhao et al., 2021; Zheng et al., 2023). The abundance of proteins related to carbohydrate metabolism, such as glycolysis and tricarboxylic acid (TCA) cycle, were increased in response to P deficiency in *B. napus* (Yao et al., 2011). However, the global protein abundance landscape of vacuole Pi transporters-mediated cellular Pi homeostasis under Pi deprivation conditions remains unclear. In this study, we identified two *BnPHT5;1a* genes (*BnA09PHT5;1a* and *BnC09PHT5;1a*), which play a crucial role in cellular Pi homeostasis in shoot leaves. Based on the proteomics analyses, a comparison of global protein abundance was performed between the leaves of *BnPHT5;1a* double mutant (DM) and wild-type W10 treated with 0 μM (-P) or 250 μM (+P) KH_2_PO_4_. Consistent with previous report, P starvation induced the accumulation of proteins involved in carbohydrate metabolism in both two *B. napus* strains. More importantly, proteins associated with ATP synthesis, polysaccharide metabolic process, and alpha-Linolenic acid metabolism showed differential accumulation between *BnPHT5;1a* DM and W10. These results provide novel understanding of vacuole Pi transport-mediated cellular Pi homeostasis in *B. napus*.

## Materials and methods

### Development of *BnPHT5;1a* double mutant lines

To develop *BnPHT5;1a* double mutant (DM) lines, two target sequences of sgRNA1 (CGACTACTACGTCAAAACCA) and sgRNA2 (TGCTCCTCTGTATCACGCAA) were selected based on CRISPR-P (http://crispr.hzau.edu.cn/CRISPR2/). The vectors (pKSE401 and pCBC-DT1T2) were applied to generate the Cas9‐BnPHT5;1a construct according to the method described by Xing et al. (2014). Transformation of *B. napus* was performed using Westar 10 hypocotyl for Agrobacterium infiltration (De Block et al., 1989). Genomic DNA of the T0 and T1 lines was isolated from leaf samples by a CTAB extraction method for mutant identification. The specific primers used to amplify the fragments containing the CRISPR/Cas9 target sites are listed in **Supplementary Table 1**. The PCR products were then sequenced using the Sanger method (Tsingke). CRISPR-P2.0 identified off-target sites (http://crispr.hzau.edu.cn/CRISPR2/). The specific primers (**Supplementary Table 1**) were applied to amply the fragments surrounding putative off-target sites, and 84 independent T2 plants with both sgRNAs were analyzed for off-target analysis, and no sequence variations were detected in these regions. Two CRISPR - Cas9 lines of *BnPHT5;1a* were obtained. The shoot and root growth of them were far less than that of WT under normal Pi, however, the phenotype of the two CRISPR - Cas9 lines had no significant difference. One of them was chosen for proteomic assay in this study.

### Plant materials and growth conditions

The *B. napus* cultivar ‘Westar 10’ and *BnPHT5;1a* DM seeds were surface sterilized for 12 min using 1% NaClO and washed with sterile pure water six times. The sterilized seeds were firstly imbibed in pure water at 4°C for 2 days. Seeds were then germinated on gauze moistened with pure water. After 7 days, uniformly sized seedlings were transferred to a one-half modified Hoagland nutrient solution (Hoagland and Arnon, 1950). The nutrient solution was refreshed every four days with gradually increasing nutritional strength. It included 1/2 HS for 5 days and complete HS with 250 µM Pi for 5 days. Ten-day-old seedlings were transferred to a P-sufficient solution (+P) or P-deficient solution (-P) for 7 days. The plants were cultivated in an illuminated growth room under 22°C and 16-h-light/8-h-dark photoperiod (with a photon flux density of 300-320 μmol m^-2^ s^-1^ and a relative humidity of 60%).

### Subcellular localization

For transient expression analysis in Arabidopsis protoplasts, the full‐length coding sequence of each *BnPHT5;1a* gene was amplified from ‘Westar 10’ genome and then cloned into the PM999 vector driven by the CaMV35S promoter using the In‐Fusion HD Cloning kit (Takara Bio). Transformation of Arabidopsis mesophyll protoplasts was performed using the method of Yoo et al. (2007). After 12–16 h incubation, the transformed protoplasts were treated with pure water to release vacuoles and GFP was observed using a microscope (TCS SP8; Leica).

### Pi transport activity assay in yeast

To generate vectors for yeast expression, the *VPT1* and *BnPHT5;1a* coding regions were amplified from Col‐0 and ‘Westar 10’ and cloned into the PRS426–ADH1 vector using the In‐Fusion HD Cloning kit (PT5162‐1; Takara Bio). The PRS426–ADH1‐PHO84 and PRS426–ADH1‐ PHO91 vectors were provided (Xu et al., 2019).

These constructs were transformed independently into the yeast strain YP100. Yeast was grown in SD/‐Trp‐Ura media with 0.67% yeast nitrogen base, 2% galactose, 0.2%‐Trp‐Ura amino acids, 1.5% agar at pH 5.6 and incubated at 30°C for 3 days. Colonies were picked for further growth in SD/‐Trp‐Ura liquid media until they reached an OD600 of 0.1. The strains were collected and washed twice with sterile water and then diluted 10^‐^, 100^‐^ and 1000^‐^ fold with ddH_2_O. Yeasts in 5 µl of diluted solution were spotted on SD (YNB‐Pi)/‐Trp‐Ura media plates supplemented with 2% glucose, 30 mM KH_2_PO_4_ at 30°C for assays.

### Protein extraction

When W10 and the *BnPHT5;1a* DM exhibited obvious phenotypic difference after being cultured under two distinct Pi conditions, the plants are sampled for comparative proteomics analysis. The experimental groups were divided into four subgroups, namely -P_W10, -P_5;1a, +P_W10 and +P_5;1a. Each subgroup had three biological replicates with five plants included, and 12 samples were prepared for protein extraction. For each sample, 1 g of leaves in total (200 mg from each of the five plants) were detached and quickly ground and homogenized in liquid nitrogen, and then transferred to 50 ml pre-cooled test tubes. To each tube, 25 ml acetone, supplemented with 10% (v/v) trichloroacetic acid (TCA), and 65 mM dithiothreitol (DTT) were added to the cell powder, followed by an overnight incubation at -20°C for protein precipitation. Following overnight precipitation, the precipitate was vacuum dried and dissolved in protein lysis buffer (1% SDS, 100 mM Tris-HCl, pH 8.0 and 1% Protease Inhibitor Cocktail), then sonicated three times on ice with a high intensity ultrasonic processor (Scientz). The remaining debris was removed by centrifugation at 20,000 g for 10 min at 4°C. Finally, the supernatant was collected and the protein concentration was determined using a BCA kit (Beyotime Institute of Biotechnology, Jiangsu, China) as directed by the manufacturer.

Protein (50 μg) from each sample was separated on a 15% SDS-PAGE gel (1.0 mm thick, 80 mm wide, and 70 mm long) and stained with Coomassie blue R250 dye. Each gel lane was cut into 7 fragments and incised into about 1 mm^3^ cubes for further in-gel digestion as previously described (Yang et al., 2018). Briefly, each section was thoroughly destained, reduced, and alkylated prior to tryptic digestion (1:50 w/w) at 37°C for 20 h and further digested with trypsin (1:100 w/w) at 37°C for 4 h. After terminating the digestion with 0.1% trifluoroacetic acid (TFA), the supernatants were transferred into new microcentrifuge tubes, and the gels were sonicated twice with extraction buffer (100% acetonitrile with 0.1% TFA). Finally, the supernatants were collected and dried in a vacuum centrifuge.

### LC-MS/MS analysis

The tryptic peptides were redissolved in 0.1% (v/v) formic acid (solvent A), and then loaded onto a 50 μm × 15 cm analytical column (C18, 3 μm, Thermo Fisher Scientific). The gradient increased from 7% to 22% in solvent B (0.1% formic acid in 80% acetonitrile) over 38 min, 22% to 32% in 14 min and climbing to 100% in 4 min then holding at 100% for the last 4 min, all at a constant flow rate of 300 ml/min on an EASY-nLC 1200 UPLC system.

The peptides were subject to an NSI source followed by tandem mass spectrometry (MS/MS) in Q ExactiveTM HF (Thermo) coupled online to the UPLC. The electrospray voltage was 2.1 kV. The m/z scan range was 350 to 1800 for a full scan. Intact peptides were detected in the Orbitrap at a resolution of 60,000. Peptides were then selected for MS/MS using NCE setting as 27 and the fragments were detected in the Orbitrap at a resolution of 15,000. A data-dependent procedure that alternated between one MS scan followed by 20 MS/MS scans with 40.0 s dynamic exclusion. Automatic gain control (AGC) was set at 1E5. Fixed first mass was 110 m/z.

### Protein identification and quantification

The resulting MS/MS data were processed using Maxquant search engine (version 1.6.14) (Tyanova et al., 2016a) for protein identification and quantification. Tandem mass spectra were searched against UniProt *B. napus* (Strain:*cv*. Darmor-bz) database concatenated with reverse decoy database. Trypsin/P was specified as cleavage enzyme allowing up to 2 missing cleavages. Carbamidomethyl (Cys) was set as fixed modification, oxidation (Met) and acetylation (Protein N-terminal) were set as dynamic modifications. The mass tolerance for precursor ions was set as 20 ppm in First search and 5 ppm in Main search, and the mass tolerance for fragment ions was set as 0.02 Da. FDR was adjusted to < 1% and minimum score for peptides was set > 40. The LFQ algorithm in MaxQuant software was exploited to quantify the protein expression level. Both unique and razor peptides were used for LFQ quantification. Perseus software (version 1.6.15.0) (Tyanova et al., 2016b) and Microsoft Excel were used for statistical analysis of the data. Only proteins that were both identified in at least one biological group and quantifiable within all three biological replicates were used for relative quantification. The arithmetic mean was used to obtain the average LFQ intensity within each biological group. A two sample Student’s t-test was used for statistical evaluation, and the coefficient of variation (CV) was computed from the three biological replicates. The differential accumulated proteins (DAPs) were defined as fold change ≥ 1.5 or ≤ 0.67, p < 0.05, and CV < 20%.

### Bioinformatics analysis

To evaluate their biological significance, the DAPs were classified into biological process, molecular function, and cellular component based on gene ontology (GO) terms with Tbtools (Chen et al., 2020). KEGG pathway annotation of the DAPs was performed using the online tool KAAS (KEGG Automatic Annotation Server, https://www.genome.jp/kaas-bin/kaas_main). GO and KEGG pathway enrichment analyses were carried out with TBtools, and a p value <0.05 was considered statistically significant. The DAPs were mapped to metabolic pathways using the KEGG website (https://www.kegg.jp/).

## Results

### *BnPHT5;1a* genes are crucial for cellular Pi homeostasis in the shoot of *B. napus*

**Figure 1:**
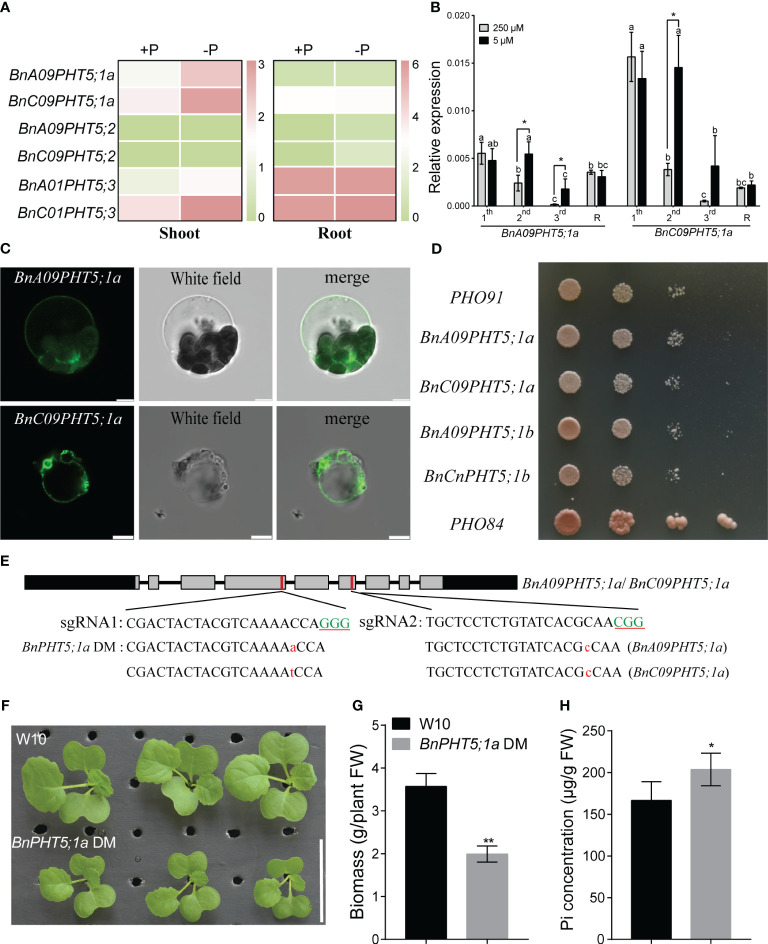
Characterization of phenotypic differences between Westar10 (W10) and *BnPHT5;1a* DM. **(A)** The transcript abundance of PHT5 members in shoot and root of W10 under phosphate (Pi) -sufficient (+P) and -deficient (-P) conditions. **(B)** The relative expression of two *BnPHT5;1a* genes in different leaves under Pi sufficient and Pi deficient conditions. **(C)** Tonoplast localization of *BnA09PHT5;1a* and *BnC09PHT5;1a*. Green fluorescent protein (GFP)‐labeled BnPHT5;1a constructs were expressed in mesophyll protoplasts from leaves of 3‐ to 4‐week‐old Arabidopsis seedlings and the GFP signal was recorded using a Leica SP8 confocal microscope. Scale bar = 5 μm. All experiments were repeated twice, and similar results were obtained. **(D)** BnPHT5;1a confers Pi transport activity in yeast. Constructs including BnPHT5;1a were expressed individually in YP100 (Δpho84Δpho87Δpho89Δpho90Δpho91Δgit1). Strains were diluted to OD600 values of 1, 0.1, 0.01 and dripped on YNB medium (pH 5.6) supplemented with glucose and 30 mM Pi for 5 days incubation at 30°C. All experiments were repeated three times and similar results were obtained. **(E)** *BnPHT5;1a DM* with different types of mutation generated by Crispr-cas9 system. Green uppercase letters indicate the “PAM” sequence, red lowercase letters indicate “insertion”. **(F)** Growth phenotype of W10 and *BnPHT5;1a DM* under Pi sufficient conditions. **(G)** The biomass of 12-day-old *B napus* seedlings of wild-type and *BnPHT5;1a DM*. Data are presented as means ± standard deviation (S.D.) (n=4). **(H)** Pi concentration of shoot in W10 and *BnPHT5;1a* DM under Pi sufficient condition. +P, Pi-sufficient condition (250 μM P); -P, Pi-deficient condition (0 μM P); S, shoot; R, root. Tukey’s test was used to analyze the statistical significance, and p < 0.05 is considered to be statistically significant. Asterisks indicate significant differences from the control, *, p<0.05; **, p<0.01.

In *BnPHT5;1* subfamily, the protein sequence similarity among BnA09PHT5;1a, BnC09PHT5;1a, BnA09PHT5;1b and BnCnPHT5;1b is over 96% (**Supplementary Table 2**) implying that BnPHT5;1a proteins may be vacuole Pi transporters. Under low Pi stress, *BnPHT5;1a* genes had stronger up-regulatory responses than those of the *BnPHT5;1b* genes in both young and mature leaves (**Figures 1A, B**), suggesting that *BnPHT5;1a* genes play crucial roles in physiological Pi status for these tissues. To validate the cellular location of *BnA09PHT5;1a* and *BnC09PHT5;1a*, green fluorescent protein (GFP) labeled BnA09PHT5;1a and BnC09PHT5;1a were constructed and their subcellular localization was analyzed in Arabidopsis protoplasts. As shown in **Figure 1C**, the GFP signals were observed on the tonoplast released from Arabidopsis protoplasts, indicating that BnA09PHT5;1a and BnC09PHT5;1a proteins were present in the vacuolar membrane. Yeast assay also indicated that BnPHT5;1a and BnPHT5;1b proteins may have similar Pi transport activity (**Figure 1D**). Therefore, the *BnPHT5;1a* genes were focused in this study and the *BnPHT5;1a* double mutant (both *BnA09PHT5;1a* and *BnC0PHT5;1a*) was generated by the CRISPR-cas9 mediated gene-editing system (**Figure 1E**). The *BnPHT5;1a* DM showed significantly retarded growth compared with wild-type *B. napus* W10 (**Figure 1F**). In contrast, W10 had lower Pi concentration in the shoot compared to *BnPHT5;1a* DM under Pi-sufficient conditions (**Figures 1G, H**). This result indicates that *BnPHT5;1a* genes are vacuolar Pi transporters and crucial for cellular Pi homeostasis in the shoot of *B. napus*.

### Protein profiles in WT and *BnPHT5;1a* DM leaves under Pi fluctuation

**Figure 2:**
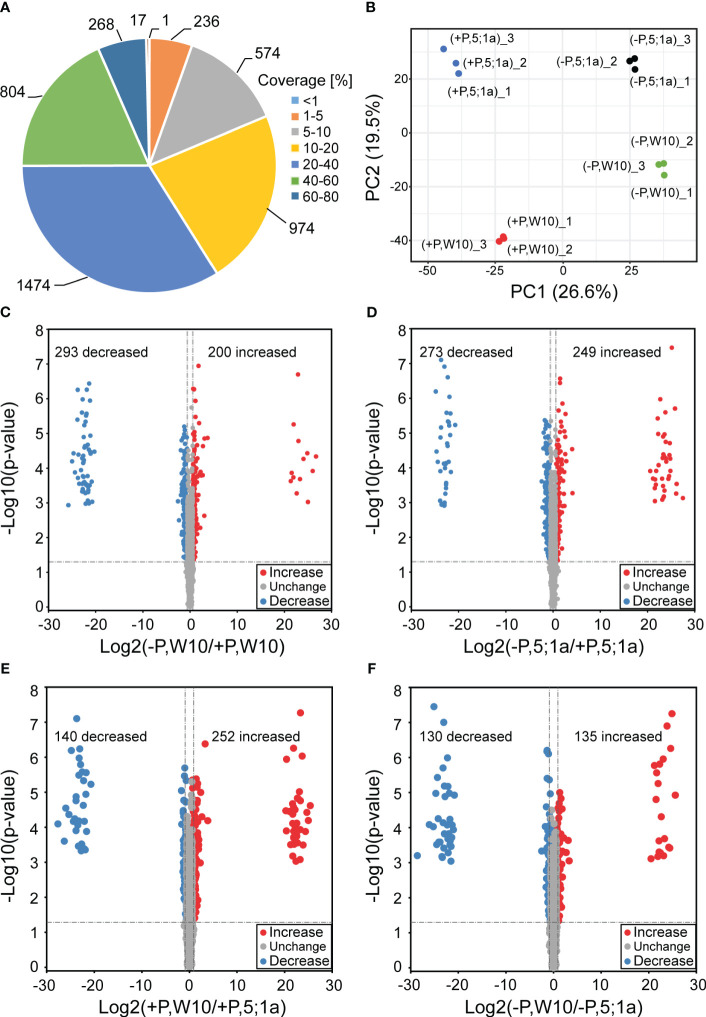
Comparative analysis of differentially accumulated proteins between *BnPHT5;1a* DM and Westar 10 (W10) under P -sufficient (+P) and -deficient (-P) conditions. **(A)** Distribution of protein sequence coverage. **(B)** Principal component analysis based on protein abundance data in the leaf samples of *BnPHT5;1a* DM and W10 under +P and –P conditions. **(C-F)** Volcano plots showing *p*-values (−log10) vs. the protein ratios (log2) between different comparison groups. Proteins with *p* < 0.05 and fold change >1.5 are considered to be differentially accumulated. The proteins with increased abundance are labeled as red dots and the proteins with decreased abundance are labeled as blue dots. +P, P-sufficient condition (250 μM); -P, P-deficient condition (0 μM).

W10 and the *BnPHT5;1a* DM exhibited the greatest phenotypic disparity after being cultured under two distinct Pi conditions for seven days (**Figure 1**). To estimate the importance of *BnPHT5;1a* genes in *B. napus* adaptation to Pi fluctuation, label-free quantitative proteomics were conducted to analyze the mature leaves of W10 and *BnPHT5;1a* DM. Seven days after treatment under Pi -sufficient (+P, 250 μM Pi) or -deficient (–P, 0 μM Pi) conditions, the mature leaves of W10 and *BnPHT5;1a* DM were collected for protein extraction (**Supplementary Figures 1**, **2**). After being analyzed by MaxQuant software, 5,756 proteins in *B. napus* were identified from the raw data, of which 4,348 proteins were detected with at least two unique peptides (**Supplementary Table 3**) and the majority of identified proteins had sequence coverage up to 60% (**Figure 2A**). Correlation analysis among the three biological replicates in each sample group revealed that quantitative results were highly reproducible (**Supplementary Figure 3**). The samples were examined through principal component analysis (PCA) based on protein quantification data. All biological replicates were grouped together in the PCA, indicating high reproducibility levels (**Figure 2B**). Furthermore, the PCA separated samples from W10 and *BnPHT5;1a* DM based on PC2, and samples from +P and –P conditions determined on PC1, indicating that W10 and *BnPHT5;1a* DM have very different responses to Pi availability, and Pi deficiency has a significant impact on the leaf proteome in *B. napus*.

A cut-off value of 1.5-fold change in either direction and p < 0.05 were used for comparative quantification analysis. The DAPs of W10 and *BnPHT5;1a* DM under the -P and +P conditions were identified, respectively. In W10, the abundance of 493 proteins were differentially accumulated, among which the abundance of 200 proteins were increased and that of 293 proteins were decreased under -P condition (**Figure 2C**; **Supplementary Figure 4A**; **Supplementary Table 4**). In *BnPHT5;1a* DM, 522 proteins showed differential accumulation, among which the abundance of 249 proteins were increased and that of 273 proteins were decreased under the -P condition (**Figure 2D**; **Supplementary Figure 4A**; **Supplementary Table 4**). Under +P conditions, 392 DAPs were detected between W10 and *BnPHT5;1a* DM (**Figure 2E**; **Supplementary Figure 4A**; **Supplementary Table 4**) including 252 proteins with increased abundance and 140 proteins with decreased abundance. Under -P conditions, 265 DAPs were detected including 135 proteins with increased abundance and 130 proteins with decreased abundance (**Figure 2F**; **Supplementary Figure 4A**; **Supplementary Table 4**). The Venn diagrams showed the distribution of shared proteins among the four comparison groups (**Supplementary Figure 4A**). Five proteins, namely BnaC07g32940D, BnaC05g04860D, BnaA01g23700D, BnaA02g08330D and caleosin gene family 3 (CLO3), displayed the pattern of increased abundance in all four comparison groups, while two proteins, BnaA03g48990D and BnaC03g34430D, displayed the pattern of decreased abundance (**Supplementary Figure 4B**).

### Gene ontology (GO) enrichment analysis of DAPs suggests energy synthesis and metabolism are suppressed in *BnPHT5;1a* DM

**Figure 3:**
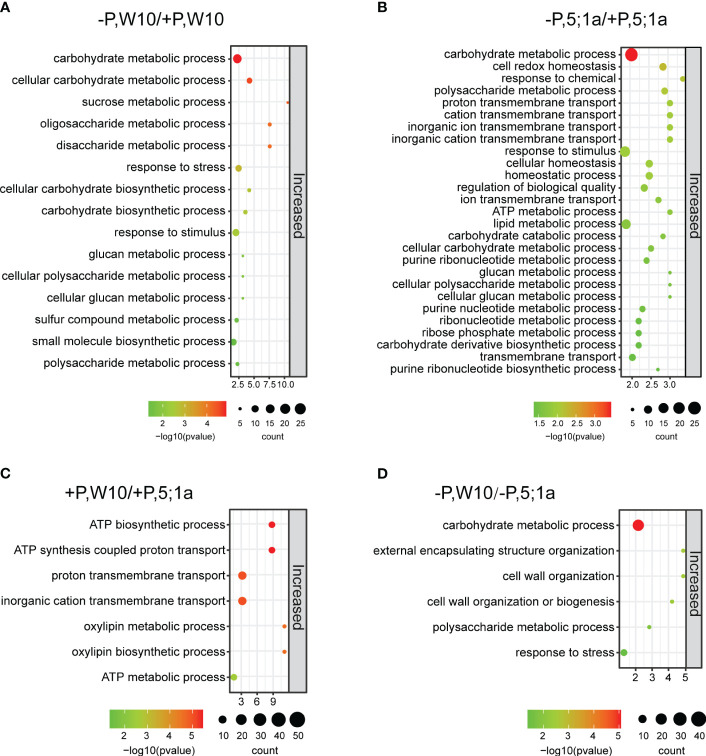
GO enrichment analysis of differentially accumulated proteins between BnPHT5;1a DM and Westar 10 (W10) under P -sufficient (+P) and -deficient (-P) conditions. **(A, B)** Enriched GO terms for proteins with increased abundance under –P condition as compared to +P condition in W10 and BnPHT5;1a DM respectively. **(C, D)** Enriched GO terms for proteins with increased abundance in W10 as compared to BnPHT5;1a DM under +P and –P conditions respectively. The results of GO enrichment analysis are shown as bubble plots. Size of the bubble indicates the number of significant proteins in the given enriched term. Color indicates the -log10 (p-value). +P, P-sufficient condition (250 μM); -P, P-deficient condition (0 μM).

We further performed Gene Ontology (GO) enrichment analyses of these identified DAPs to understand their biological significance (**Figure 3**, **Supplementary Figures 5**, **6**). In W10, DAPs were involved mainly in carbohydrate metabolic process, response to stimulus, sucrose metabolic process, cellular polysaccharide metabolic process and cellular glucan metabolic process (**Figure 3A**; **Supplementary Table 5**). In *BnPHT5;1a* DM, DAPs showed similar GO enrichment observed in W10, such as carbohydrate metabolic process, polysaccharide metabolic process, and response to stimulus; besides, they were also associated with cell redox homeostasis, proton transmembrane transport and lipid metabolic process (**Figure 3B**; **Supplementary Table 5**). Noteworthy, the number of proteins with increased abundance in *BnPHT5;1a DM* was greater than in W10, which might reflect complementary effects of other proteins to the lack of BnPHT5;1a proteins.

Under +P conditions, the proteins with increased abundance in W10 were enriched mainly in ATP biosynthetic and metabolic processes as compared to *BnPHT5;1a* DM (**Figure 3C**), suggesting that *BnPHT5;1a* DM has reduced ability in ATP synthesis and metabolism than W10; while the proteins with decreased abundance were shown to be involved in porphyrin-containing compound metabolic process and translation (**Supplementary Table 5**). Almost all of the W10 over-accumulated proteins in the category of ATP synthesis were members of the ATP synthase, which contained ATP synthase subunit delta (BnaCnng66500D, BnaA09g23090D), ATP synthase subunit gamma (BnaC04g43510D, BnaC03g29960D), F1F0 ATP synthase (BnaA01g34180D), ATP synthase protein MI25 (BnaCnng12890D) and ATP synthase subunit d (BnaA04g05550D) (**Supplementary Figure 7A**). Under -P conditions, the proteins with increased abundance in W10 mainly pointed to carbohydrate metabolism (**Figure 3D**), which is related to carbohydrate metabolism for energy supply; the proteins with decreased abundance were particularly related to proteolysis (**Supplementary Table 5**). These results suggest that Pi fluctuation mainly disturbs the carbohydrate metabolic process in *B. napus* and loss of function in *BnPHT5;1a* genes severely causes the same response in normal Pi conditions. Besides, more physiological processes were associated with the function of *BnPHT5;1a* genes.

### KEGG pathway enrichment analysis of DAPs

**Figure 4:**
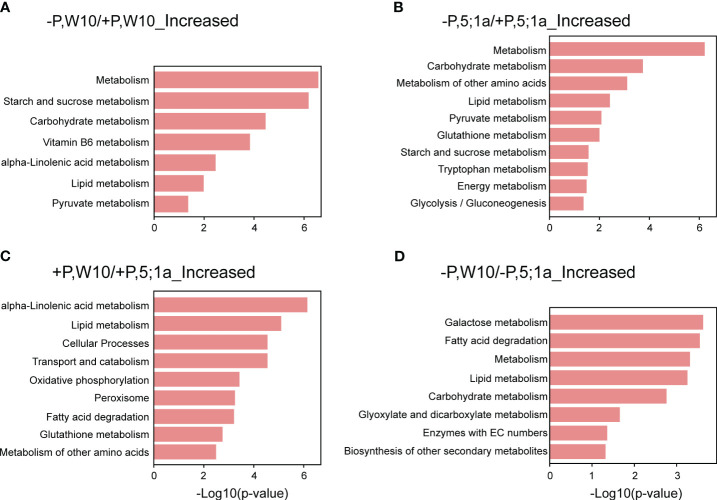
KEGG pathway enrichment analysis of differentially accumulated proteins between BnPHT5;1a DM and Westar 10 (W10) under P -sufficient (+P) and -deficient (-P) conditions. **(A, B)** Enriched KEGG pathways for proteins with increased abundance under –P condition as compared to +P condition in W10 and BnPHT5;1a DM respectively. **(C, D)** Enriched KEGG pathways for proteins with increased abundance in W10 as compared to *BnPHT5;1a* DM under +P and –P conditions respectively. The results of KEGG pathway enrichment analysis are shown as horizontal bar plots. The abscissa corresponds to the -log10 (*p-*value), and the ordinate represents the enriched KEGG pathways. +P, P-sufficient condition (250 μM); -P, P-deficient condition (0 μM).

KEGG pathway enrichment analysis revealed the metabolic pathways for the proteins with increased abundance in W10 and *BnPHT5;1a* DM under Pi deficiency, respectively (**Figures 4A, B**; **Supplementary Table 6**). As a whole, the proteins with increased abundance under -P in both W10 and *BnPHT5;1a* DM were enriched in biological pathways related to nutrient and energy metabolism. Specifically, the -P induced proteins in W10 were mainly involved in the metabolism of starch, sucrose, vitamin B6, alpha-Linolenic acid metabolism (ALA), lipids and pyruvate. The proteins with increased abundance under -P in *BnPHT5;1a* DM were also involved in the metabolism of lipid, pyruvate, starch and sucrose; besides, they were found to relate to glutathione metabolism, tryptophan metabolism and glycolysis/gluconeogenesis. The shared pathways overrepresented by the proteins with decreased abundance under Pi deficiency in W10 and *BnPHT5;1a* DM include translation and ribosome (**Supplementary Table 6**).

**Figure 5:**
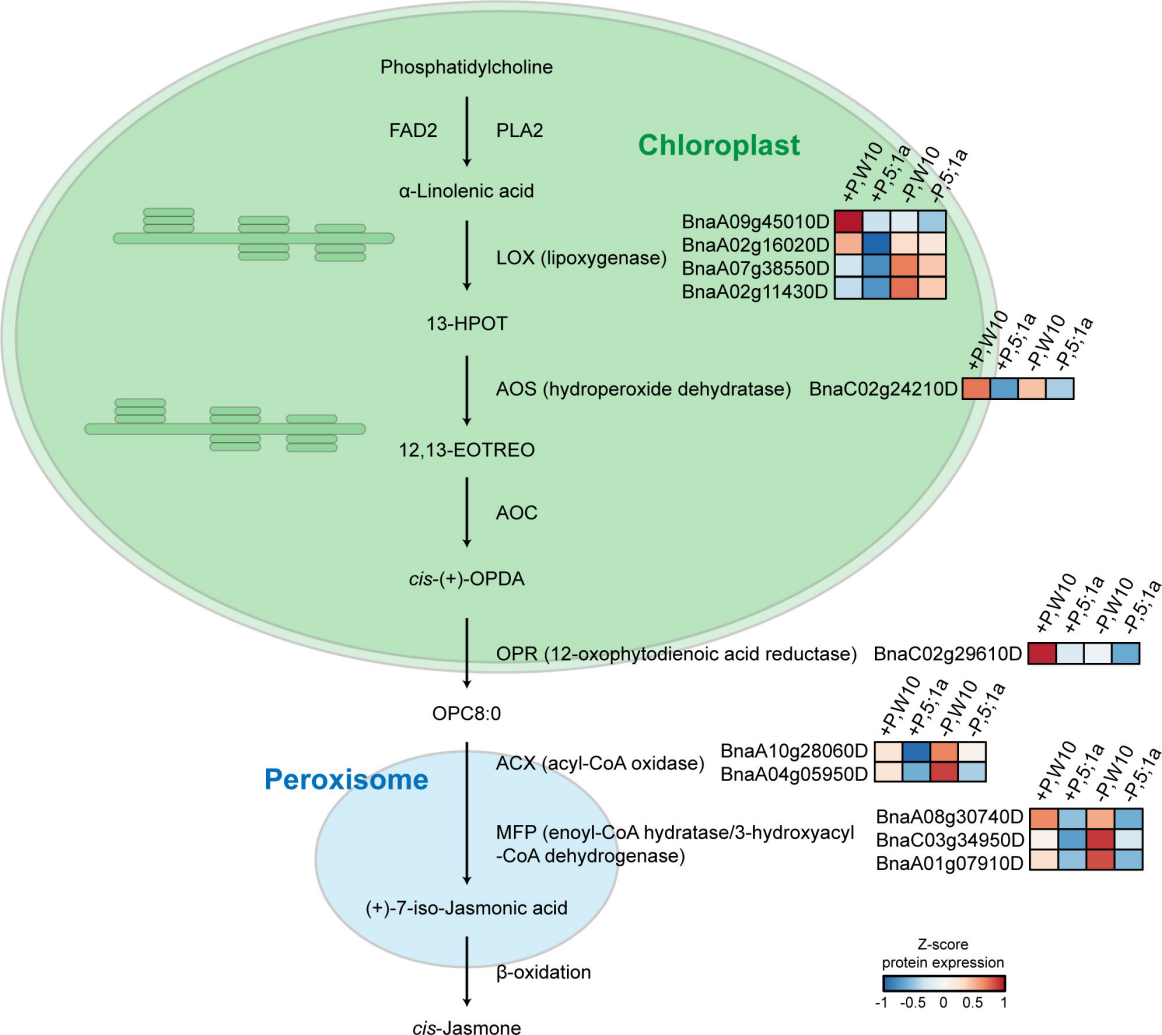
Proteins with higher abundance in W10 as compared to *BnPHT5;1a* DM under P-sufficient (+P) and P-deficient (-P) conditions in the pathway of alpha-Linolenic acid (ALA) metabolism. Heat maps with different colors indicate the relative protein abundance value.

Under +P conditions, most of the enriched pathways for the proteins with increased abundance in W10 as compared to *BnPHT5;1a* DM were also mainly related to metabolism, most notably ALA metabolism, followed by lipid metabolism, glutathione metabolism and metabolism of other amino acids (**Figure 4C**; **Supplementary Table 6**). In addition, oxidative phosphorylation, the biological pathway that generates ATP, was also highly enriched in W10, which was consistent with the above GO enrichment results (**Figures 3C**, **4C**). Pathways overrepresented by the proteins with decreased abundance in W10 as compared to *BnPHT5;1a* DM include porphyrin metabolism and metabolism of cofactors and vitamins (**Supplementary Table 6**). In the pathway of ALA metabolism, 11 of the 28 associated proteins showed higher abundance in W10 than that in *BnPHT5;1a* (**Figure 5**; **Supplementary Figure 7B**). Due to the complex genome of allotetraploid, these 11 proteins were attributed to five functional proteins, which were lipoxygenase (BnaA09g45010D, BnaA02g16020D, BnaA07g38550D, BnaA02g11430D), hydroperoxide dehydratase (BnaC02g24210D), 12-oxophytodienoic acid reductase (BnaC02g29610D), acyl-CoA oxidase (ACX, BnaA10g28060D, BnaA04g05950D) and enoyl-CoA hydratase/3-hydroxyacyl-CoA dehydrogenase (MFP2, BnaA08g30740D, BnaC03g34950D, BnaA01g07910D). Phosphatidylcholine serves as the source for the production of ALA, and could be further transformed into different metabolites through distinct metabolic pathways. Proteins with higher abundance in W10 participated in the production of two major metabolites, the 10-OPDA and jasmonate (JA) (**Figure 5**). As 10-OPDA is a key product of JA synthesis, ALA metabolism in W10 might contribute to the synthesis of more JA than in *BnPHT5;1a*.

Under -P conditions, the enriched pathways for the proteins with increased abundance in W10 as compared to *BnPHT5;1a* DM include galactose metabolism, fatty acid degradation, lipid metabolism, carbohydrate metabolism, glyoxylate and dicarboxylate metabolism, and the biosynthesis of other secondary metabolites; while few pathways were overrepresented by the proteins with decreased abundance (**Figure 4D**; **Supplementary Table 6**). The proteins with increased abundance involved in carbohydrate metabolism comprise galactinol-sucrose galactosyltransferase, α-galactosidase, β-galactosidase, malate dehydrogenase, citrate synthase, formate dehydrogenase, aldehyde dehydrogenase, glutamine synthetase, pectinesterase and Xyloglucan endotransglucosylase/hydrolase (XTH) (**Supplementary Figure 7C**). In summary, compared with *BnPHT5;1a* DM, the proteins with increased abundance in W10 were significantly associated with the metabolism of various substances, especially the metabolism of energy-supplying substances, which was also in consistent with the GO enrichment results. Therefore, the mutation of *BnPHT5;1a* may affect the cellular Pi homeostasis in the shoot, thus altering the abundance of proteins involved in energy synthesis and metabolism, and finally impacting the growth and development of *BnPHT5;1 DM* at the seedling stage.

## Discussion

### *BnPHT5;1a* is required for Pi homeostasis regulation in *B. napus*

Our previous study has identified *BnPHT5;1b* as a vacuolar Pi influx transporter in *B. napus*. *BnPHT5;1a* is the closest homologue of *BnPHT5;1b*; however, unlike *BnPHT5;1b* (Han et al., 2022), the expression of *BnPHT5;1a* was significantly induced in young and mature leaves of *B. napus* under Pi starvation (**Figure 1B**), suggesting their different roles in Pi homeostasis regulation in these tissues. Subsequent to the knockout of *BnPHT5;1a* in *B. napus*, growth inhibition was observed, concomitant with an elevation in leaf P concentration, indicating *BnPHT5;1a*’s involvement in Pi transport and allocation across the whole rapeseed plant. Moreover, under normal P conditions, ablation of *BnPHT5;1a* led to a reduction in ATP synthesis capacity (**Figure 3C**; **Supplementary Figure 7A**), implying its function as a vacuolar phosphorus transporter within cells, influencing the distribution of phosphorus within vacuoles and ATP-generating organelles (such as mitochondria and chloroplasts).

### Proteins related to carbohydrate metabolism and cell wall organization are crucial for *BnPHT5;1a*-mediated Pi homeostasis in *B. napus*

Previous studies have reported the increased accumulation of proteins related to carbohydrate metabolism in response to Pi deficiency in *B. napus*. Enhancement of carbon metabolism is a powerful way for plants to cope with various abiotic stresses (Pommerrenig et al., 2018; Saddhe et al., 2021). Here, we found that Pi deprivation increased the abundance of proteins involved in carbohydrate metabolism in the mature leaves of both W10 and *BnPHT5;1a* DM strains. Interestingly, proteins related to carbohydrate metabolism in W10 showed overall higher abundance than those in *BnPHT5;1a* DM (**Supplementary Figure 7C**), which indicated the partial impairment of carbohydrate metabolism in *BnPHT5;1a* DM under the -P condition. Thus, *BnPHT5;1a* is important for the carbohydrate metabolism of *B. napus* upon Pi deprivation.

Under Pi deficiency, there is typically an augmentation in cell wall porosity, which enhances the absorption of both water and nutrients (Liao et al., 2011). Pectinesterase and Xyloglucan endotransglucosylase/hydrolase (XTH) are involved in carbohydrate metabolism and cell wall organization. They can exploit polysaccharides in the cell wall to provide a carbon source for the TCA cycle (Wu et al., 2018; Ishida and Yokoyama, 2022), thereby loosening the cell wall and accelerating organic acid secretion. Overexpression of XTH confers plants the ability to adapt to a wide range of environmental stresses, such as salt stress (Xu et al., 2020), cold stress (Takahashi et al., 2021) and aluminium toxicity (Du et al., 2021). In W10, the abundance of Pectinesterase and XTH were much higher than that in *BnPHT5;1a* DM under the –P condition (**Supplementary Figure 7C**). Thus, BnPHT5;1a proteins are critical for the low-P induced accumulation of Pectinesterase and XTH, as well as a significant increase in cell wall porosity for Pi uptake.

### Ablation of *BnPHT5;1a* may lead to decreased citrate and malate production in *B. napus*

Citrate and malate can be secreted from root cells into the rhizosphere soil when Pi is limited. Their excretion will promote Pi solubilization in the soil and contribute to more efficient Pi uptake by plants (Tomaz et al., 2010; Plaxton and Tran, 2011). Overexpression of citrate synthase in tobacco and Arabidopsis could increase their Pi-uptake ability under low-P stress (Koyama et al., 2000; López-Bucio et al., 2000). Besides, citrate and malate function as metabolic intermediates of the TCA cycle that support ATP synthesis (Sweetlove et al., 2010). Malate dehydrogenase (MDH) and citrate synthase are two key enzymes mediating citrate and malate synthesis (Journet et al., 1981). The abundance of MDH and citrate synthase in W10 mature leaves were higher than those in *BnPHT5;1a* DM under –P conditions. This indicates that W10 may produce more citrate and malate than *BnPHT5;1a* DM to maintain energy supply in response to a sudden drop in Pi availability. It should be noted that some of the DAPs in our study, such as MDH and citrate synthase (**Supplementary Figure 7C**), showed similar changes to those observed in transcriptomic and proteomic studies conducted in rice (Wasaki et al., 2003) and maize (Li et al., 2008) under -P conditions, lending credence to the reliability of our quantitative proteomics data.

### ALA metabolism and jasmonate biosynthesis are likely involved in *BnPHT5;1a*-mediated Pi homeostasis in *B. napus*

In W10, ALA metabolism was the most overrepresented pathway compared to *BnPHT5;1a* DM under sufficient Pi supply (**Figure 4C**, **Supplementary Figure 7B**), which suggested attenuated ALA metabolism in *BnPHT5;1a* DM. In ALA metabolism, phosphatidylcholine acts upstream of ALA and is the precursor of ALA (Bae et al., 2022). Phosphatidylcholine is the major phospholipid found in the membranes of plant cells and its biosynthesis needs Pi (Liu et al., 2021; Nakamura, 2021). Under Pi starvation, phosphatidylcholine can also be hydrolyzed and contributes to Pi supply for cell metabolism (Li et al., 2006). Thus, ALA metabolism is highly correlated with Pi homeostasis through phosphatidylcholine metabolism. In *BnPHT5;1a* DM, attenuated ALA metabolism may further destabilize Pi homeostasis.

JA biosynthesis is the major pathway downstream of ALA metabolism. Nearly all W10 proteins enriched in the ALA metabolism pathway participate in JA biosynthesis (**Figure 5**). JA is a type of phytohormone that alleviates a variety of abiotic stresses in plants, such as cold stress, boron stress and cadmium stress (Dar et al., 2015). Recent evidence has shown the crosstalk between JA signaling and Pi deficiency stress. In Arabidopsis (*Arabidopsis thaliana*), tomato (*Solanum lycopersicum*) and tobacco (*Nicotiana benthamiana*), Pi deficiency could induce JA biosynthesis to enhance their resistance to insect herbivory (Khan et al., 2016). In rice (*Oryza sativa*), JA could increase the root and shoot soluble Pi content by regulating Pi reutilization in the root cell walls and Pi translocation from root to shoot, which could alleviate the inhibitory effect of P deficiency on shoot biomass (Tao et al., 2022). According to the proteomics data, JA biosynthesis was likely more active in W10 compared with *BnPHT5;1a* DM (**Figure 5**), suggesting its important role in *BnPHT5;1a*-mediated Pi homeostasis in *B. napus*.

### Shared proteins can be potential markers of Pi homeostasis in *B. napus*

It is worth noting that the abundance of five proteins was all increased and that of two proteins all decreased in the four comparison subgroups (**Supplementary Figure 4**), indicating that they were directly related to the function of *BnPHT5;1a* and low phosphorus stimulation. It has been reported that mutation of *VPT1* in *Arabidopsis*, and *BnPHT5;1b* in *B. napus*, impaired Pi homeostasis of the plants. Here, the difference in the phenotype, and the accumulation of proteins, especially between +P W10 and +P *BnPHT5;1a* group, suggest that mutation in *BnPHT5;1a* DM impaired Pi homeostasis in *B. napus*.

The five proteins with increased abundance were BnaC07g32940D, BnaC05g04860D, BnaA01g23700D, BnaA02g08330D and CLO3. Among them, BnaC07g32940D is a UBX domain-containing protein with potential function in proteasome-mediated ubiquitin-dependent protein degradation process. Under Pi deficient-conditions, two RING-finger ubiquitin E3 ligases SDEL1 and SDEL2 trigger SPX4 (SPX-domain-containing proteins) degradation, which releases Pi central regulators, PHOSPATE STARVATION RESPONSE proteins (PHRs) to activate Pi starvation response in rice (Ruan et al., 2019). It is reasonable that the accumulation of BnaC07g32940D might associate with critical process of Pi homeostasis in *B. napus.* BnaC05g04860D, also named as 2-oxoadipate dioxygenase/decarboxylase, is involved in a D-lysine catabolic pathway (Thompson et al., 2020). BnaA01g23700D is a dienelactone hydrolase domain-containing protein. BnaA02g08330D, a calcium-transporting ATPase, catalyzes the hydrolysis of ATP coupled with the transport of calcium. CLO3 is a calcium-binding protein and can be strongly induced in leaves of *Arabidopsis thaliana* by various abiotic stress conditions including salt, dehydration, and abscisic acid treatment (Takahashi et al., 2000). Calcium, an important intracellular second messenger, has recently been described as also functioning in signaling Pi status in *Arabidopsis thaliana* (Matthus et al., 2019).

Among the two proteins with decreased abundance, BnaA03g48990D is a cytoplasmic protein functioning in organelle organization, whereas BnaC03g34430D possesses pectin acetylesterase activity and is supposed to be involved in cell wall organization. The mutation of *BnPHT5;1a* decreased the accumulation of these two proteins, which altered cellular architecture under Pi deprivation.

Taken together, all these seven shared proteins could be the potential markers of Pi homeostasis in *B. napus*, although their function needs further investigation in the future.

## Conclusions

In this study, we have identified and characterized two vacuolar Pi transporter genes *BnA09PHT5;1a* and *BnC09PHT5;1a*. Disruption of *BnPHT5;1a*s impaired cellular Pi status in *B. napus*. According to a comparative proteomic analysis, *BnPHT5;1a* is likely to be involved primarily in ATP synthesis, carbohydrate metabolism, sugar-associated PSI regulation, cell wall organization, and ALA/JA-mediated Pi distribution. Additionally, seven new protein markers of Pi homeostasis are identified in *B. napus.* These findings suggest that vacuolar Pi transporter probably functions as a balancer in mature leaves, regulating cellular Pi equilibrium during Pi deprivation.

## Data availability statement

The datasets presented in this study can be found in online repositories. The names of the repository/repositories and accession number(s) can be found below: ProteomeXchange, PXD047074.

## Author contributions

BH: Writing – review & editing, Writing – original draft, Visualization, Validation, Software, Resources, Project administration, Methodology, Investigation, Formal Analysis, Data curation, Conceptualization. JY: Visualization, Validation, Software, Resources, Project administration, Methodology, Investigation, Formal analysis, Data curation, Writing – review & editing, Writing – original draft. TW: Writing – review & editing. XY: Writing – review & editing. YW: Writing – review & editing. GD: Writing – review & editing. JH: Writing – review & editing, Writing – original draft. CW: Writing – review & editing. FX: Writing – review & editing. SW: Writing – review & editing, Writing – original draft. LS: Writing – review & editing, Writing – original draft, Supervision, Funding acquisition, Conceptualization.

## Supplementary material

Supplementary Figure 1 Strategies for in-gel fractionation of the whole protein extracts of shoot from W10 and *BnPHT5;1a* DM under P -sufficient (+P) and deficient (-P) conditions. Equal amounts of proteins (50 µg) each sample were separated on a 15% SDS-PAGE gel and stained by coomassie brilliant blue R250 dye. Each lane was fractionated into seven pieces for further proteomic analysis. Three biological replicates of each treatment were performed independently. +P, P-sufficient condition (250 μM); -P, P-deficient condition (0 μM).

Supplementary Figure 2 Workflow of the label-free quantitative proteomic experiment. 7-day-old seedlings of wild-type Westar 10 (W10) and *BnPHT5;1a* double mutants (DM) were cultured under two distinct Pi conditions for 7 days. Then leaves from each sample were collected for in-gel digestion-based label-free quantitative proteomic analysis. +P, P-sufficient condition (250 μM); -P, P-deficient condition (0 μM); 5;1a, *BnPHT5;1a* DM.

Supplementary Figure 3 Pairwise correlation of protein label-free quantitative (LFQ) intensities derived from MaxQuant software among three biological replicates.

Supplementary Figure 4
**(A)** Venn diagrams of differentially accumulated proteins (DAPs) with increased abundance (left) and decreased abundance (right). **(B)** The abundance profiles of shared DAPs. +P, P-sufficient condition (250 μM); -P, P-deficient condition (0 μM). 5;1a, *BnPHT5;1a DM*. Heat maps with different colors indicate the relative protein abundance value.

Supplementary Figure 5 Bubble plot showing the enriched “cellular component (CC)” and “molecular function (MF)” GO terms of differentially accumulated proteins under –P condition as compared to +P condition in **(A)** W10 and **(B)** *BnPHT5;1a* DM. Size of the bubble indicates the number of significant proteins in the given enriched term. Color indicates the -log_10_(p) value. +P, P-sufficient condition (250 μM); -P, P-deficient condition (0 μM).

Supplementary Figure 6 Bubble plot showing the enriched “cellular component (CC)” and “molecular function (MF)” GO terms of differentially accumulated proteins between W10 and *BnPHT5;1a* DM under **(A)** +P and **(B)** -P conditions. Size of the bubble indicates the number of significant proteins in the given enriched term. Color indicates the -log_10_(p) value. +P, P-sufficient condition (250 μM); -P, P-deficient condition (0 μM).

Supplementary Figure 7 Difference in the abundance profiles of proteins in selected biological pathways between BnPHT5;1a DM and W10 under P -sufficient (+P) and -deficient (-P) conditions. **(A)** ATP synthesis; **(B)** ALA metabolism; **(C)** carbohydrate metabolism.

Supplementary Table 1 Primers used in this study.

Supplementary Table 2 Amino acid similarity matrix for proteins in *BnPHT5;1* subfamily.
