# Supplementary material for: Proteomics reveals the significance of vacuole Pi transporter in the adaptability of Brassica napus to Pi deprivation
Source: Front Plant Sci. 2024 Mar 25;15:1340867. doi: 10.3389/fpls.2024.1340867 (PMC11000671; doi:10.3389/fpls.2024.1340867)
Supplement: Supplementary Figure 1 — Strategies for in-gel fractionation of the whole protein extracts of shoot from W10 and BnPHT5;1a DM under P -sufficient (+P) and deficient (-P) conditions. Equal amounts of proteins (50 µg) each sample were separated on a 15% SDS-PAGE gel and stained by coomassie brilliant blue R250 dye. Each lane was fractionated into seven pieces for further proteomic analysis. Three biological replicates of each treatment were performed independently. +P, P-sufficient condition (250 μM); -P, P-deficient condition (0 μM). [file DataSheet_1.docx]

**Supplementary Information for**

**Proteomics reveals the significance of vacuole Pi transporter in the adaptability of *Brassica napus* to Pi deprivation**

Bei Han^1,2^, Junjun Yan^1^, Tao Wu^1,3^, Xinyu Yang^1,3^, Yajie Wang^1,3^, Guangda Ding^1,3^, John Hammond^4^, Chuang Wang^3^, Fangsen Xu^1,3^, Sheliang Wang^1,3^, Lei Shi^1,3^

1. National Key Laboratory of Crop Genetic Improvement, Huazhong Agricultural University, Wuhan 430070, China

2. Zhejiang University-Hangzhou Global Scientific and Technological Innovation Center, Zhejiang University, Hangzhou, Zhejiang 311215, China

3. Microelement Research Center, College of Resources & Environment, Huazhong Agricultural University, Wuhan, 430070, China

4. School of Agriculture, Policy and Development, University of Reading, Reading RG6 6AR, UK

**
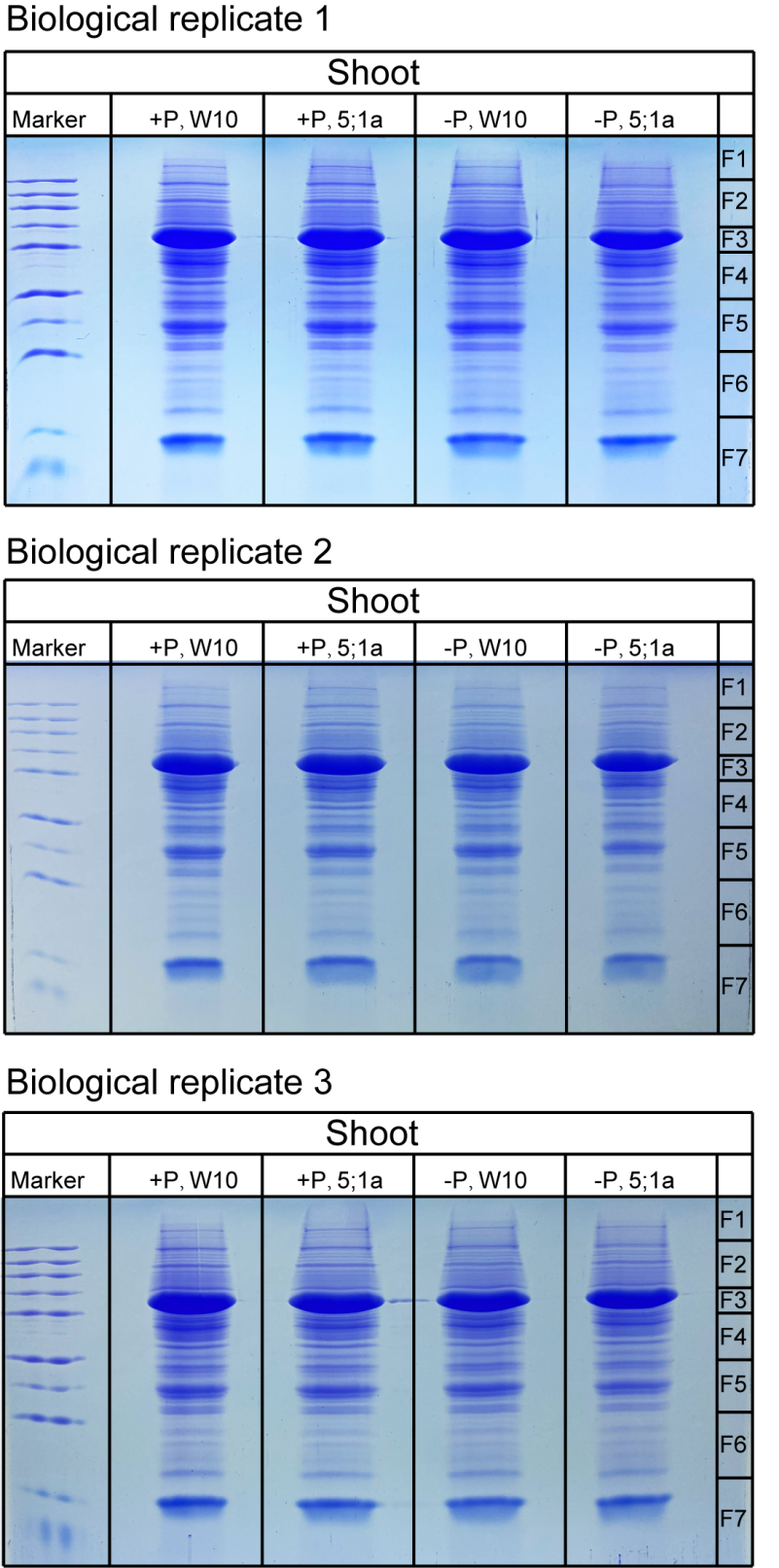
Supplemental Figure 1. Strategies for in-gel fractionation of the whole protein extracts of shoot from W10 and *BnPHT5;1a* DM under P -sufficient (+P) and deficient (-P) conditions.** Equal amounts of proteins (50 µg) each sample were separated on a 15% SDS-PAGE gel and stained by coomassie brilliant blue R250 dye. Each lane was fractionated into seven pieces for further proteomic analysis. Three biological replicates of each treatment were performed independently. +P, P-sufficient condition (250 μM); -P, P-deficient condition (0 μM).

**
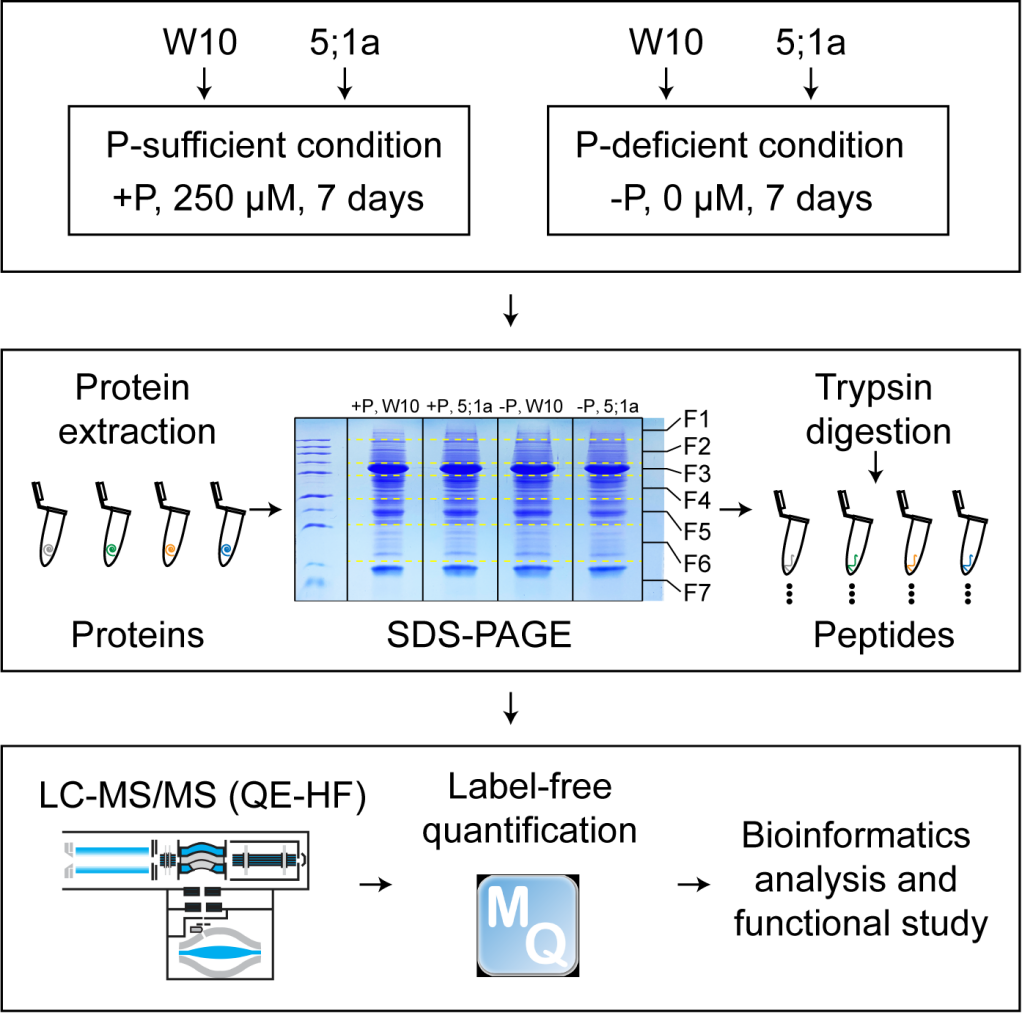
Supplemental Figure 2. Workflow of the label-free quantitative proteomic experiment.**

7-day-old seedlings of wild-type Westar 10 (W10) and *BnPHT5;1a* double mutants (DM) were cultured under two distinct Pi conditions for 7 days. Then leaves from each sample were collected for in-gel digestion-based label-free quantitative proteomic analysis. +P, P-sufficient condition (250 μM); -P, P-deficient condition (0 μM); 5;1a, *BnPHT5;1a* DM.

**
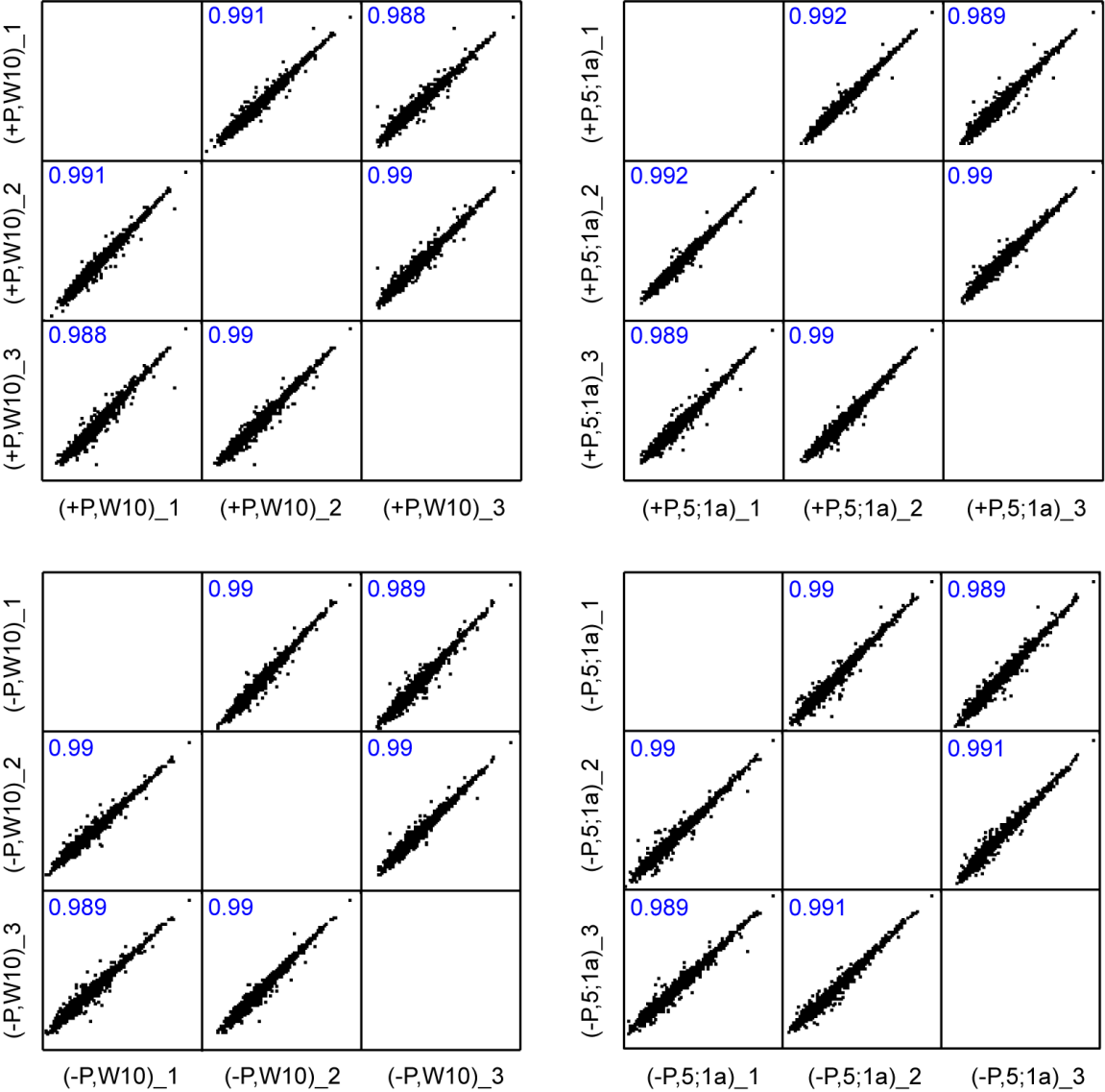
Supplemental Figure 3. Pairwise correlation of protein label-free quantitative (LFQ) intensities derived from MaxQuant software among three biological replicates.**

**
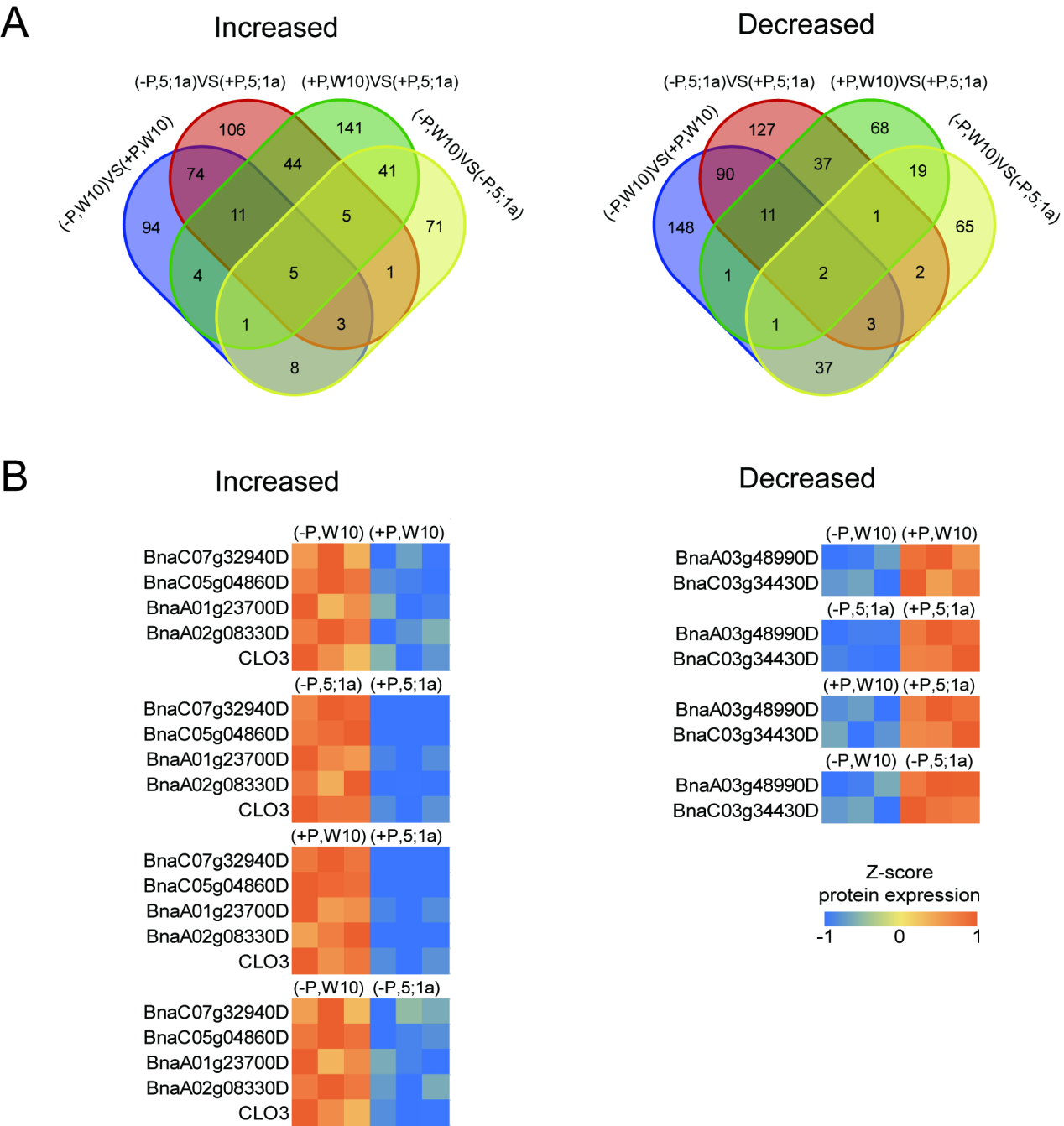
Supplemental Figure 4. (A) Venn diagrams of differentially accumulated proteins (DAPs) with increased abundance (left) and decreased abundance (right) . (B) The abundance profiles of shared DAPs.** +P, P-sufficient condition (250 μM); -P, P-deficient condition (0 μM). 5;1a, *BnPHT5;1a DM*. Heat maps with different colors indicate the relative protein abundance value.

**
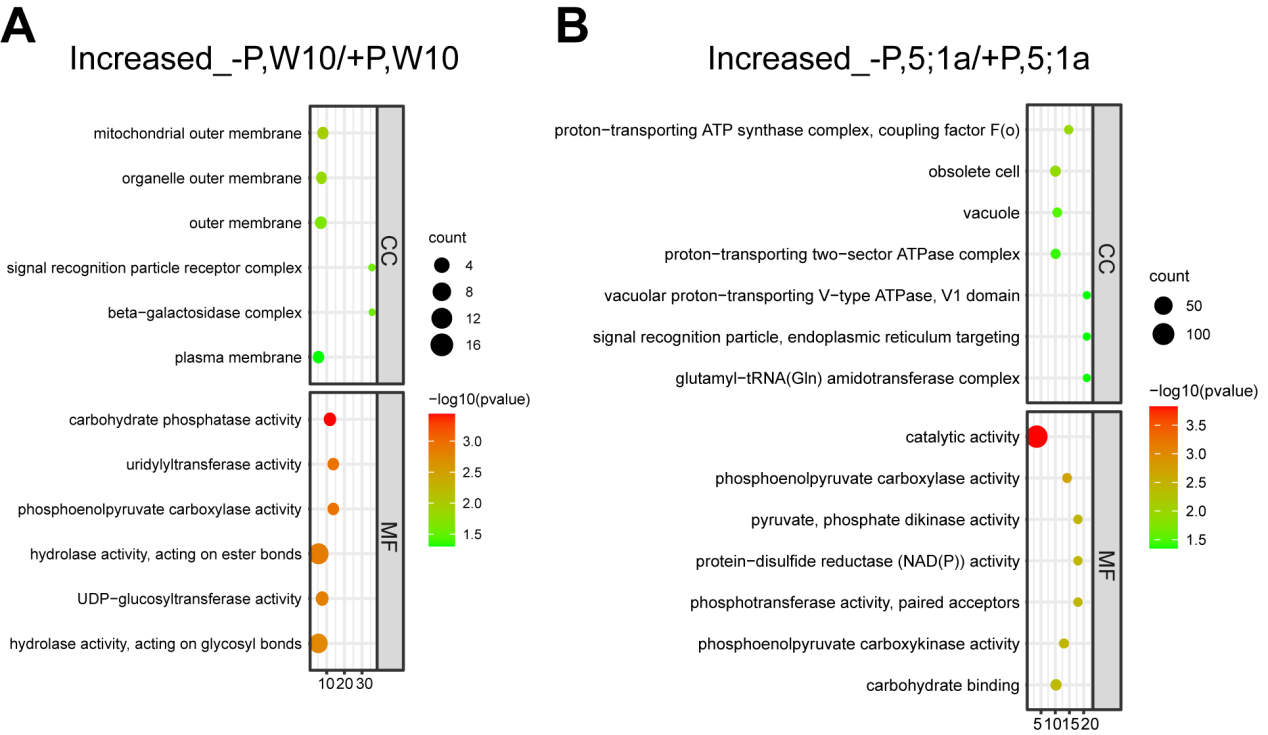
Supplemental Figure 5. Bubble plot showing the enriched “cellular component (CC)” and “molecular function (MF)” GO terms of differentially accumulated proteins under –P condition as compared to +P condition in (A) W10 and (B) *BnPHT5;1a* DM.** Size of the bubble indicates the number of significant proteins in the given enriched term. Color indicates the -log_10_(p) value. +P, P-sufficient condition (250 μM); -P, P-deficient condition (0 μM).

**
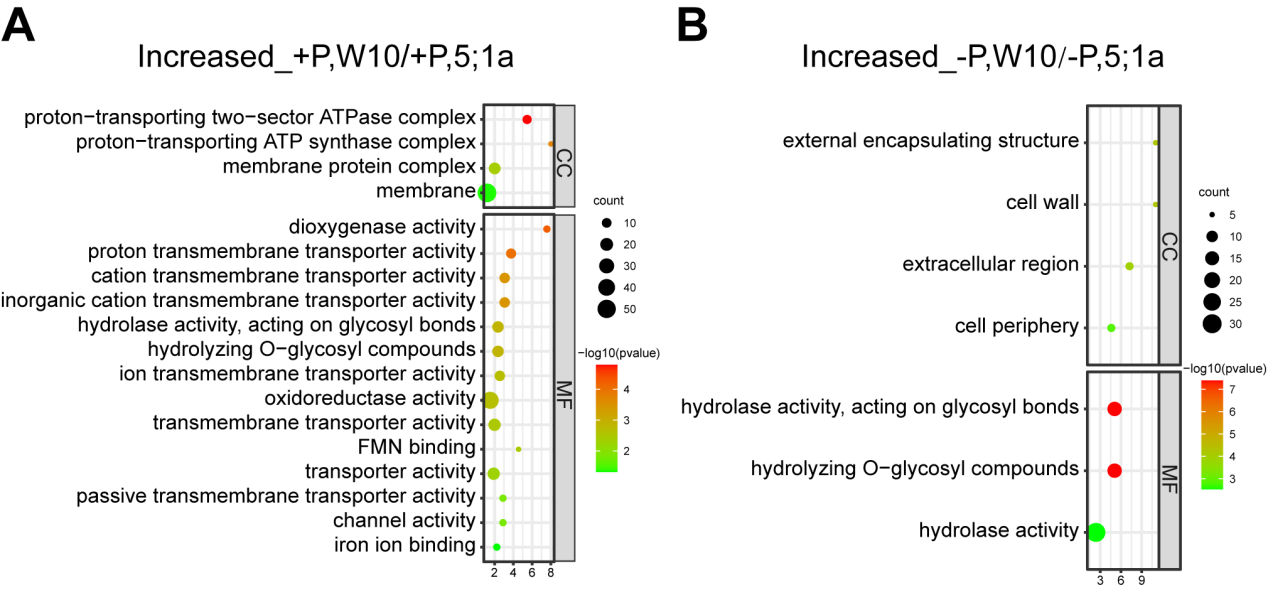
Supplemental Figure 6. Bubble plot showing the enriched “cellular component (CC)” and “molecular function (MF)” GO terms of differentially accumulated proteins between W10 and *BnPHT5;1a* DM under (A) +P and (B) -P conditions.** Size of the bubble indicates the number of significant proteins in the given enriched term. Color indicates the -log_10_(p) value. +P, P-sufficient condition (250 μM); -P, P-deficient condition (0 μM).

**
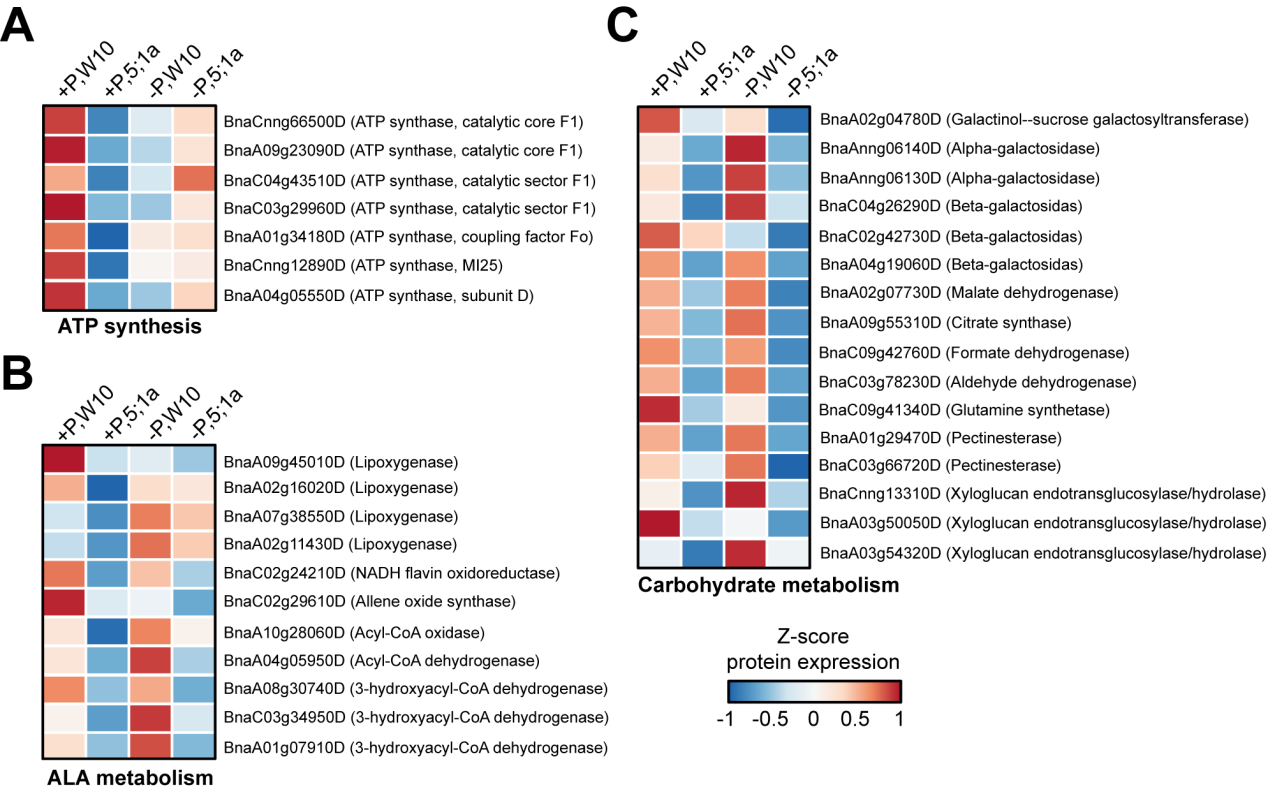
Supplemental Figure 7. Difference in the abundance profiles of proteins in selected biological pathways between** BnPHT5;1a **DM and W10 under P -sufficient (+P) and -deficient (-P) conditions.** (A) ATP synthesis; (B) ALA metabolism; (C) carbohydrate metabolism.

**SUPPLEMENTAL TABLES**

**Supplemental Table 1. Primers used in this study.**

| **Primers** | **sequence** |
| --- | --- |
| **Primers for CRISPR/Cas9 vector construction** | |
| H Cas9PHT5;1a-DT1-BsF 1 | ATATATGGTCTCGATTGGACTACTACGTCAAAACCAGTT |
| Cas9PHT5;1a-DT1-F0 | TGGACTACTACGTCAAAACCAGTTTTAGAGCTAGAAATAGC |
| Cas9PHT5;1a-DT2-R0 | AACTTGCGTGATACAGAGGAGCCAATCTCTTAGTCGACTCTAC |
| Cas9PHT5;1a-DT2-BsR | ATTATTGGTCTCGAAACTTGCGTGATACAGAGGAGCCAA |
| **Primers for CRISPR target sites sequencing** | |
| Cas9-A09PHT5;1a-F | CTGCTCGGAGGAAGATTATCCACGCTGAGGGAAACT |
| Cas9-A09PHT5;1a-R | CTCTGTTTGTCACTGCATATCATCCAAGAGAG |
| Cas9-C09PHT5;1a-F | TTGCTGGCAGGGAGATTATCCACGCTGAGGGAAACG |
| Cas9-C09PHT5;1a-R | TTCATTCAAGAGAATCCAATCAAAT |

**Supplemental Table 2. Amino acid similarity matrix for proteins in *BnPHT5;1* subfamily.**

|  | **BnA09PHT5;1a** | **BnC09PHT5;1a** | **BnA09PHT5;1b** | **BnCnPHT5;1b** |
| --- | --- | --- | --- | --- |
| **BnA09PHT5;1a** |  | 99.00% | 97.05% | 96.63% |
| **BnC09PHT5;1a** | 99.00% |  | 97.60% | 97.46% |
| **BnA09PHT5;1b** | 97.05% | 97.60% |  | 99.58% |
| **BnCnPHT5;1b** | 96.63% | 97.46% | 99.58% |  |
